# Supplementary material for: Sortilin limits EGFR signaling by promoting its internalization in lung cancer
Source: Nat Commun. 2017 Oct 30;8:1182. doi: 10.1038/s41467-017-01172-5 (PMC5662760; doi:10.1038/s41467-017-01172-5)
Supplement: Supplementary file 1 — Supplementary Information [file 41467_2017_1172_MOESM1_ESM.pdf]

Supplementary Figure 1

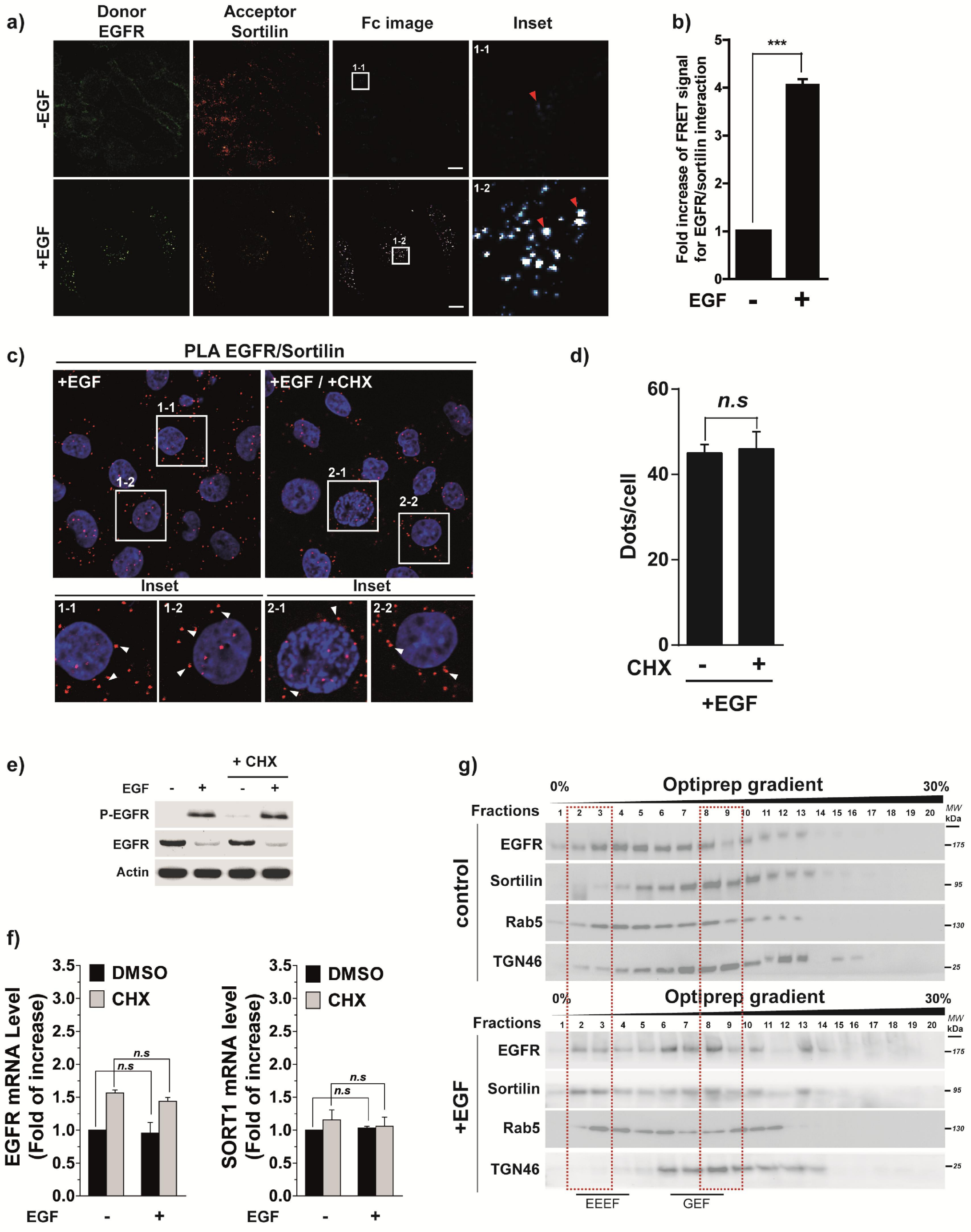

Supplementary Figure 1 (related to Figure 1) (a) A549 cells stimulated or not with EGF (50 ng/mL) for 30 min were incubated with anti-EGFR (donor) and anti-sortilin (acceptor). Next, fluorescence resonance energy transfer (FRET) was analyzed by confocal microscopy. **(b)** FRET is quantified relative to non-stimulated A549 cells. **(c)** A549 cells were pretreated or not with the protein synthesis inhibitor cycloheximide (CHX) (100 µg/mL) for 2 h, and then stimulated with EGF (50 ng/mL^{-1}) for 30 min. Cells were fixed, and proximity ligation assays were performed using anti-EGFR and anti-sortilin antibodies. Red dots indicate sites of EGFR–sortilin interaction (white arrows). Scale bar, 10 µm. **(d)** PLA quantification is shown relative to EGF-stimulated A549 cells. **(e)** A549 cells were pretreated or not with CHX (100 µg/mL) for 2 h, and then stimulated or not with EGF (50 ng/mL) for 30 min. The cell lysates were analyzed by western blotting for P-EGFR and EGFR. **(f)** Quantitative PCR analysis of EGFR and sortilin (*SORT1*) expressions, under the same conditions described above for **(e)**. Results are presented in terms of fold change after normalizing against *HPRT* mRNA. **(g)** Post-nuclear Supernatant from A549 cells treated or not with EGF (50 ng/mL) for 30 min were loaded onto a 0–30% OptiPrep density gradient medium and subjected to ultracentrifugation. Early endosome (Rab5) and Golgi (TGN46) markers were used to identify the corresponding fractions. EGFR and sortilin levels in the respective organelle-enriched fractions were analyzed by immunoblotting. All values represent means ± SD, Student's t-test ***P<0.001. Each experiment has been repeated at least three times.

Supplementary Figure 2

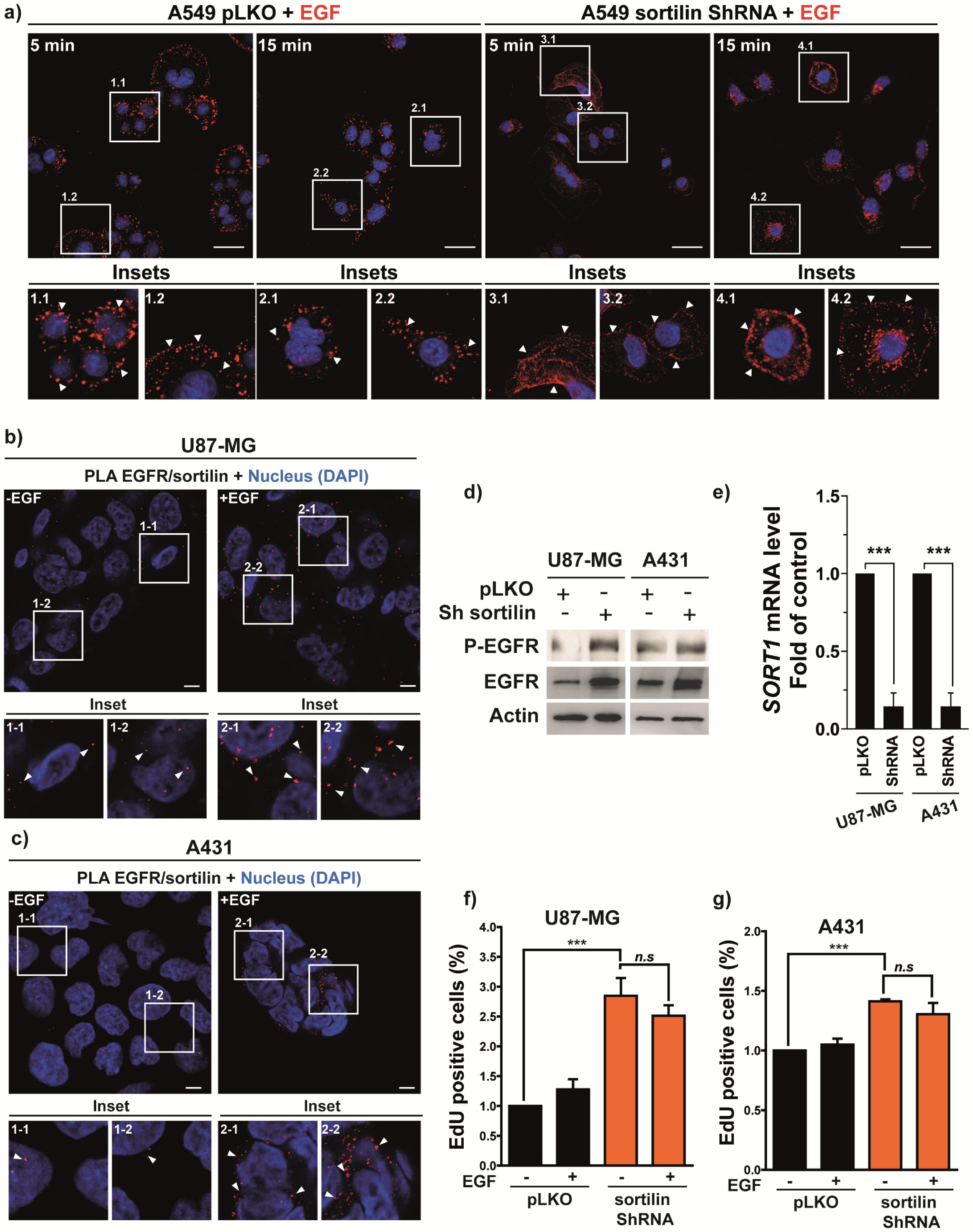

Supplementary Figure 2 (related to Figure 4) (a) Representative images for A549 control and sortilin-depleted cells following stimulation with fluorescent EGF. Scale bar, 10 µm. **(b,c)** Proximity ligation assays (PLA) were performed on U87-MG and A431 cells stimulated or not with EGF (50 ng/mL) for 30 min. Red dots indicate the sites of PLA amplification, reflecting the EGFR–sortilin interaction (white arrows). Scale bar, 10 µm. **(d)** Lysates from sortilin-depleted U87-MG and A431 (sortilin shRNA) cells and control cells (pLKO), analyzed by western-blotting for P-EGFR and EGFR. **(e)** Validation of sortilin depletion in U87-MG and A431 cells by quantitative PCR to detect sortilin (SORT1) mRNA. Results are presented in terms of fold change after normalization against HPRT mRNA. **(f,g)** Representative histograms of cell proliferation, as determined by EdU incorporation. Control or sortilin-depleted U87-MG and A431 cells were stimulated with EGF (50 ng/mL) for 1 h, and then fixed and processed for EdU incorporation. The percentages of EdU-positive cells were calculated by flow cytometry. All values represent means ± SD, Student's t-test ***P<0.001. Each experiment has been repeated at least three times.

Supplementary Figure 3

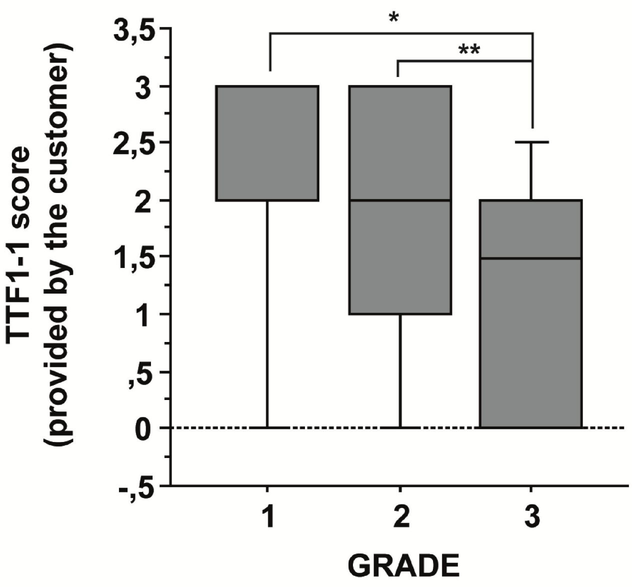

Supplementary Figure 3 (related to figure 6). Boxplot diagram represents quantification (score provided by the manufacturer, US Biomax) of TTF1 expression in human lung adenocarcinoma (*n=78*), and revealed that its expression decreases with increasing pathologic grade. All values represent means ± SD, Student's t-test ***P<0.001. Each experiment has been repeated at least three times.
